# Relationship between types and levels of free fatty acids, peripheral insulin resistance, and oxidative stress in T2DM: A case-control study

**DOI:** 10.1371/journal.pone.0306977

**Published:** 2024-08-12

**Authors:** Hamidreza Shiri, Hossein Fallah, Moslem Abolhassani, Saba Fooladi, Zohreh Ramezani Karim, Behnaz Danesh, Mojtaba Abbasi-Jorjandi

**Affiliations:** 1 Department of Clinical Biochemistry, Faculty of Medicine, Tehran University of Medical Sciences, Tehran, Iran; 2 Applied Cellular and Molecular Research Center, Kerman University of Medical Sciences, Kerman, Iran; 3 Endocrinology and Metabolism Research Center, Institute of Basic and Clinical Physiology Sciences, Kerman, Iran; 4 Physiology Research Center, Kerman University of Medical Sciences, Kerman, Iran; 5 Student Research Committee, Kerman University of Medical Sciences, Kerman, Iran; 6 Department of Internal Medicine, Afzalipour School of Medicine, Kerman University of Medical Sciences, Kerman, Iran; Muhimbili University of Health and Allied Sciences School of Medicine, UNITED REPUBLIC OF TANZANIA

## Abstract

Free Fatty Acids (FFAs) are vital for energy homeostasis and the pathogenesis of a variety of diseases, including diabetes. For the first time, we presumed and investigated the types and levels of FFAs and their links to Insulin Resistance (IR) and Oxidative Stress (OS) in T2DM. A case-control study was conducted on 60 individuals with diabetes, 60 prediabetics with IFG, and 60 control groups. A Gas Chromatography Flame Ionization Detector (GC-FID) was used to estimate FFAs, which were then classified based on length and saturation. Indeed, antioxidant parameters such as TAC, MDA levels, PON-1, SOD-3, and CAT activity were assessed. Higher levels of LCFFA, SFFA, USFFA, and total FFA were found in people with diabetes and prediabetes. These levels were also linked to higher levels of HOMA-IR, BMI, FBS, HbA_1_C, and MDA, but lower levels of antioxidants. Furthermore, adjusting the above FFAs with age, sex, and antihypertensive medication increased T2DM development. SCFFA and ω3/6 fatty acids had a negative relationship with HOMA-IR, FBS, and insulin and a positive relationship with TAC. Adjusted SCFFA reduces T2DM risk. According to our models, total FFA is utilized to diagnose diabetes (AUC = 83.98, cut-off > 919 μM) and SCFFA for prediabetes (AUC = 82.32, cut-off < 39.56 μM). Total FFA (≥ 776 μM), LCFFA (≥ 613 μM), SFFA (≥ 471 μM), and USFFA (≥ 398 μM) all increase the risk of T2DM by increasing OS, BMI, and HOMA-IR. On the other hand, SCFFAs (≥ 38.7 μM) reduce the risk of T2DM by reducing BMI, HOMA-IR, and OS. SCFFAs and total FFAs can be used for the diagnosis of prediabetes and diabetes, respectively.

## Introduction

### Highlights

**Fig 1:**
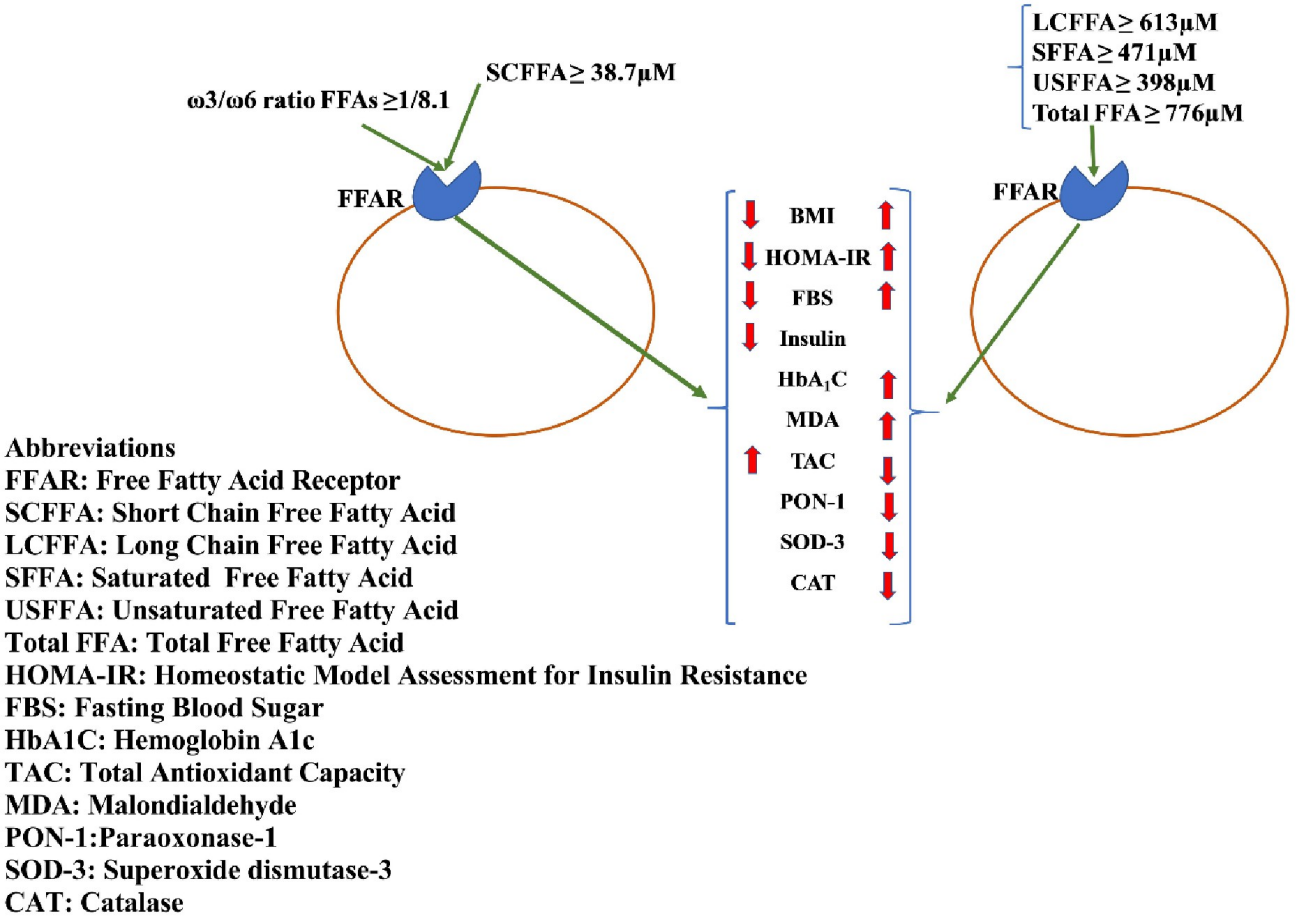
Graphical abstract.

- Based on Fig 1, SCFFAs (≥ 38.7 μM) are associated with a reduced risk of T2DM by reducing BMI, HOMA-IR, and OS.
- Total FFA (≥ 776 μM), LCFFA (≥ 613 μM), SFFA (≥ 471 μM), and USFFA (≥ 398 μM) are all associated with increased risk of T2DM by increasing OS, BMI, and HOMA-IR.
- Total FFA (≥ 919.9 μM) and SCFFAs (≤ 39.56 μM) can efficiently serve as diagnostic markers for diabetes and prediabetes, respectively.

Type 2 diabetes mellitus (T2DM) affects 90% of diabetic individuals, and this will reach 700 million by 2045 [1], and in 2025, 5.2 million people in Iran will be diabetic (9.6%) [2]. Prediabetes is a disorder characterized by Impaired Fasting Glucose (IFG) and Impaired Glucose Tolerance (IGT) which raises the risk of developing T2DM [3]. Prediabetes is a stage between normal and diabetes and increases the odds of T2DM. Indeed, patients with prediabetes may not have any symptoms and not require medication, while diabetes can cause symptoms and require medication [4]. More studies have been done on diabetic patients, and few studies have been done on prediabetic patients. Metformin is the first-line therapy for diabetes, and it improves insulin sensitivity by activating AMP Dependent Kinase (AMPK) [5]. The main causes of diabetes are still unknown and need further study. However, obesity, a sedentary lifestyle, defects in insulin signaling pathways, inflammation, the accumulation of lipids and fatty acids, and oxidative stress (OS) are all considered risk factors for it [6, 7].

Free Fatty Acids (FFAs) are divided into different types based on their length and chemical structure [8]. A recent study has revealed that in addition to the total plasma level of FFAs, changes in the type and levels of FFAs are important in regulating the body’s physiological and pathological processes [9]. Free Fatty Acid Receptors (FFARs) are expressed in different parts of the body, and they are affected by different types of FFAs and induce different effects [10]. A temporary rise in FFAs in pancreas causes insulin secretion, but a permanent increase leads to the dysfunction of β-cell and ultimately death of cells and induces diabetes [11]. Among FFA, Palmitic Acid (PA) has proven to cause increase Glycated hemoglobin A_1_c (HbA_1_c) [12]. Several hypotheses have been proposed to explain insulin resistance (IR) due to FFAs and PA in muscle, hepatocyte, and adipose tissue, such as the Randall cycle, Toll-Like Receptor (TLR) stimulation, inflammation, activation of Protein Kinase C (PKC), accumulation of harmful lipids such as diacylglycerol, impaired mitochondrial function, and Oxidative Stress (OS) induction [13, 14]. Furthermore, decreasing omega 3 (ω3) and omega 9 (ω9) and increasing ω6 fatty acids have the main effect on diabetes severity [15, 16]. All of the studies surveyed total FFA or single FFA, such as PA [17]. For example, cohort studies by Li et al. showed a total FFA increase in diabetes and total FFA development risks of T2DM incidence [18]. Studies by Spiller et al. showed a total FFA increase in T2DM [19]. This study used a variety of free fatty acids. These included short-chain free fatty acids (SCFFA < 8 carbons), medium-chain free fatty acids (MCFFA 8–12 carbons), long-chain free fatty acids (LCFFA > 12 carbons), unsaturated free fatty acids (USFFA with at least one unsaturated bond), saturated free fatty acids (SFFA without a double bond), ω3 and ω6 fatty acids, and total free fatty acids (total FFAs) [20].

OS is characterized by an inconsistency in the formation of Reactive Oxygen Species (ROS) and a deficiency in the antioxidant defense system [21]. ROS stimulates c-Jun N-terminal kinase (JNK), NF-κB, and inflammation pathways, which in turn interfere with insulin signaling and induce IR [22]. Paraoxonase-1 (PON-1) is a calcium-dependent enzyme esterase that binds to HDL-c and has antioxidant and anti-atherosclerosis roles [23]. The enzyme catalase (CAT) is the main regulator of hydrogen peroxide (H_2_O_2_) metabolism [24]. Superoxide dismutases (SOD) are metalloenzymes at the first line of defense of antioxidant enzymes against ROS and superoxide (O^-^_2_) [24]. Total Antioxidant Capacity (TAC) represents the total activity of all antioxidant groups in plasma [25]. One of the outcomes of excess ROS is the destruction of fatty acids. This is a process in which free radicals attack the double bonds of fatty acids, producing malondialdehyde (MDA), hexanal, and 4-hydroxynonenal [26]. Studies have demonstrated FFAs can disrupt the process of oxidative phosphorylation and lead to an increase in ROS production, OS, and an increased inflammatory response [27]. However, the type and levels of FFA, as well as their effect on OS, have yet to be investigated.

Because FFAs play an important role in the development of many diseases, we decided to test for the first time the type and levels of FFAs based on length and saturation in diabetics and prediabetics and see how they related to HOMA-IR, OS, and biochemical parameters.

## Methods and materials

### Subjects and data collection

This case-control study was performed on diabetics in early stages treated with metformin (HbA1c > 6.5, and FBS > 125 mg/dl, n = 60), prediabetics, people with IFG with HbA1c levels (5.7 ≤ HbA1c ≤ 6.5) and FBS levels (100 mg/dl ≤ FBS ≤ 125 mg/dl) which is in this range with no therapeutic intervention (n = 60) [28], and control groups (n = 60) in Kerman, Iran, from 2020 to 2021 (Fatemeh Al-Zahra Hospital and Javad Al-A’meh Clinic). For the participants in this study, first, the purpose of the study was explained, and it was stated that the patient’s information would be kept confidential. So, the people who were eligible and willing to participate in the study and were matched in gender and age signed the written consent form and entered the study. In this form, demographic information, weight, height, antihypertensive medication, other disease, and blood pressure about participants was collected. Participants who had consumed antilipemic drugs, dietary supplements consumed one month before the study such as vitamin E, vitamin D, and vitamin C, and inflammatory diseases such as acute infections, chronic lung and liver disease, cardiovascular disease, kidney failure, and type 1 diabetes were excluded from the study under the supervision of an internal medicine physician. After collecting information from participants and overnight fasting, 10 ml of peripheral blood was collected with an aseptic procedure in plain tubes and EDTA tubes, and the serum and plasma were separated via centrifugation and stored at -70°C until further analysis. The Kerman Medical Sciences Ethics Committee (IR.KMU.REC.1397.531) approved this study, which was carried out under the *Helsinki Declaration*.

### Biochemical markers

Biochemical markers such as Fasting Blood Sugar (FBS), Total Cholesterol (TC), triglycerides (TG), High-Density Lipoprotein cholesterol (HDL-c), Alanine Transaminase (ALT), Aspartate Transaminase (AST), Creatinine (CR), Blood Urea Nitrogen (BUN), and Uric Acid (UA) levels were measured using an autoanalyzer (Selectra-XL, Vital Science; Netherlands) and specific kits (Pars Azmoon, Tehran, Iran) to analyze the samples. HbA_1_c was measured by an enzymatic method (Pishtazteb, Tehran, Iran). The insulin level was evaluated by an ELISA (Monobind, California, USA). The concentration of Low-Density Lipoprotein cholesterol (LDL-c) was calculated using the Friedewald equation, and Homeostatic Model Assessment for Insulin Resistance (HOMA-IR) was carried out using FBS (mmol) * fasting serum insulin (U/ml)/22.5.

### Paraoxonase-1 arylesterase activity

Based on Bobin-Dubigeon et al., the phenylacetate reagent (Saint Louis, MO, USA) was used to measure the PON-1 arylesterase activity in plasma [29]. This enzyme catalyzes the hydrolysis of phenylacetate to phenol and acetate. The absorbance of phenol was evaluated at 270 nm for substrate hydrolysis.

### Malondialdehyde measurement

The method used for evaluating MDA levels of Thiobarbituric Acid (TBA) was based on the test procedure by Yagi et al. [30]. Briefly, plasma and Trichloroacetic Acid (TCA) were mixed with TBA and heated in boiling water. The reaction mixture was centrifuged, and the absorbance was measured at 535 nm.

### Superoxide dismutase-3 activity

SOD-3 activity was measured using the Randox kit procedure in plasma (UK; Cat No. RS504). In this procedure, xanthine oxidase produces O_2_^-^ and H_2_O_2_, which lead to the conversion of NBT to NBT-diformazan. By decreasing O_2_^-^, SOD-3 inhibits the synthesis of NBT-diformazans. Therefore, SOD-3 activity is measured by determining the level of reduction in NBT-diformazan.

### Catalase activity

CAT activity was determined according to the protocol put forward by Hadwan in plasma [31]. A phosphate buffer, H_2_O_2_, and a dichromate/acetic acid solution were added to the reaction solution. The test tubes were heated in a 100°C oven for 8 minutes, and absorbance was evaluated at 570 nm.

### Total antioxidant capacity in plasma

The procedure used by Abolhassani et al. was slightly modified to estimate the level of Total Antioxidant Capacity (TAC) in plasma [32]. The Ferric-Reducing Ability of Plasma (FRAP) technique was employed. At 3.5 pH, the ferric tripyridyl triazine complex was converted into ferrous tripyridyl triazine.

### Measurement of FFAs with gas chromatography

We used the methods suggested by Kangani et al [33], but made a few changes to the methods, the plasma values, and an internal standard [17, 34]. In summary, the Dole solution, which is made up of HCL (1M), n-hexane, and isopropanol (1:10:40, v/v/v), was used to get lipids out of plasma. In order to keep the lipids from oxidizing during extraction, 0.05 mg/mL of butylated hydroxytoluene was added to the Dole solution. After that, 4 mL of Dole reagent was mixed with 450 μl of plasma and 50 μl of the internal standard (pentadecanoic acid, C15, 1 mg/ml). The mixture was then vortexed for 20 minutes. After a 10-minute incubation at 25°C, the test tubes were filled with 2 mL of distilled water and 4 mL of n-hexane. After 5 minutes, isolate the supernatant n-hexane from the solution, and then centrifuge at 4000 RPM for 10 minutes to extract the total lipids. n-hexane was placed inside the oven to evaporate, and then 200–300 μL of chloroform was added to the lipid phase separated in the previous step to dissolve the lipids in it. Using a solvent of acetic acid, diethyl ether, and n-hexane (1:30:70, v/v/v), Thin-Layer Chromatography (TLC) was used to separate FFAs from other lipids. Iodine vapor was used to identify FFAs on TLC plates based on standards. The FFA bands on the TLC scrape and solvent in 1 ml of chloroform and methanol (3:1) were vortexed for 30 seconds to dissolve the FFAs and centrifuged for two minutes at 4000 RPM to separate the FFAs. In the next step, FFAs were converted into Free Fatty Acid Methyl Esters (FAMEs) by boron trifluoride in methanol. The FAMEs were then separated using an Agilent GC-7890A instrument with a flame ionization detector based on retention time (RT) and the AUC (Area Under the Curve) of the FAMEs standard. The injection volume was 1μl in the splitless mode. A capillary column was operated (DB-225, 20 m×0.1 mm I.D., 0.1μm film thickness, USA). Furthermore, FAMEs were categorized in this study based on their size and degree of saturation [20].

### Statistical analysis

The quantitative data were reported in terms of the Standard Error of the Mean (SEM) while the qualitative data were reported as proportions/percentages. In order to determine data normality, the Kolmogorov-Smirnov test was used, and based on the results, appropriate parametric or non-parametric tests were used. A one-way ANOVA/Kruskal-Wallis test was used to analyze group differences, along with post-hoc Tukey/Mann-Whitney U tests and Chi-square testing. Spearman’s test was also employed to examine the type of FFAs, demographic information, BMI, and HOMA-IR correlation. Also, a linear regression test was done to investigate the association of OS, biochemical markers, and BMI (independent variables) on FFAs (dependent variables). The links between T2DM (diabetes and prediabetes) and FFAs were evaluated using multinomial logistic regression with adjustments for age, gender, and antihypertensive medication. Furthermore, quantitative data were expressed in quartiles, and the first quarter was used as a reference for comparison in determining the odds ratio (OR) of disease. The diagnostic utility of FFAs for diabetes and prediabetes was determined using the Receiver Operating Characteristic (ROC) with an AUC at 95% CI. The Youden index was used to generate cut-off values for each of the FFAs. Statistical analysis of the variables and graphs was performed using SPSS software version 23 and GraphPad Prism software, respectively. P-values less than 0.05 were considered statistically significant.

## Results

### Demographic and biochemical markers

**Fig 2:**
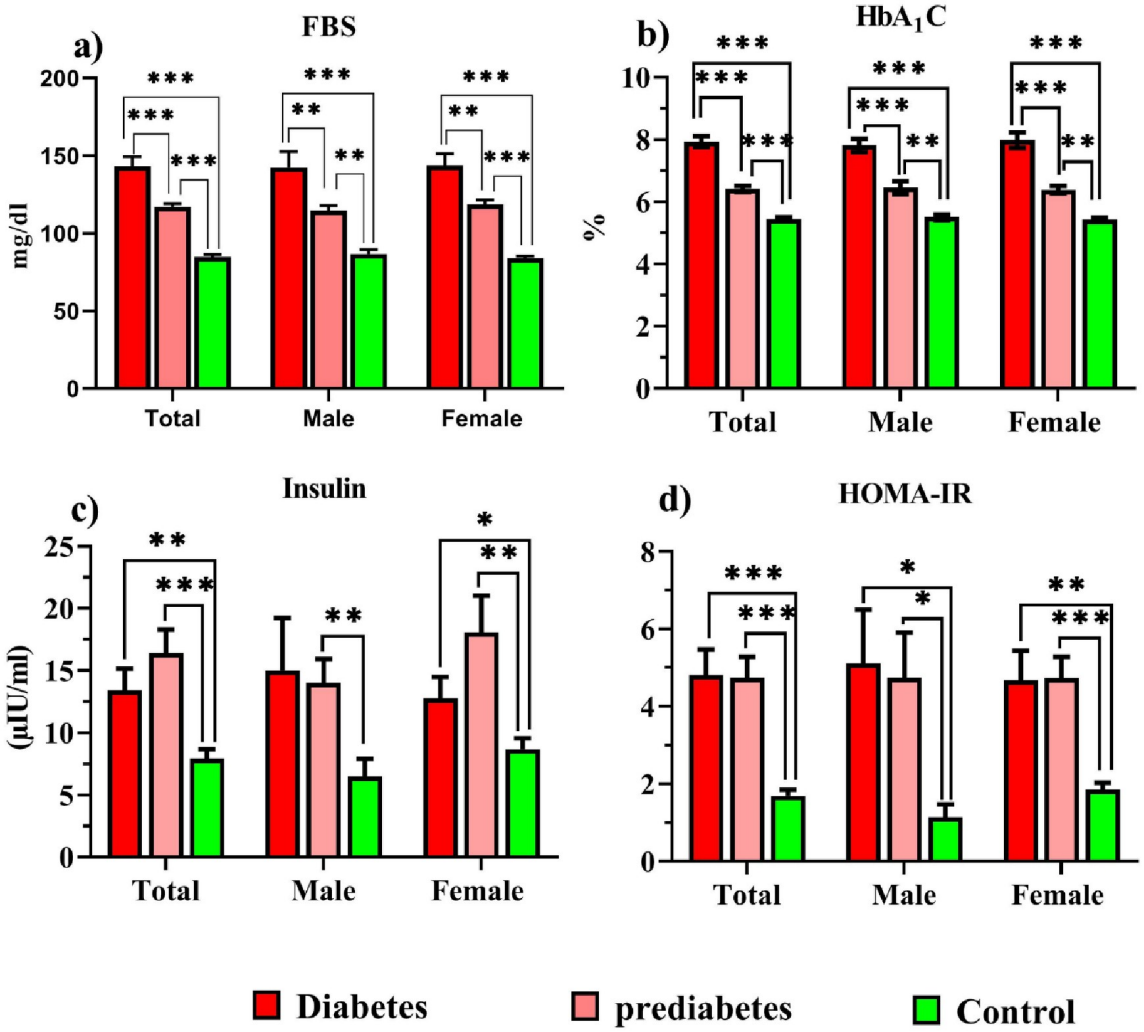
Comparison of FBS, HbA_1_c, insulin, and HOMA-IR in study groups.

Demographic data and biochemical markers are shown in S1 Table in S1 File, and Fig 2. In the diabetic and prediabetic groups, the antihypertensive medication (*P = 0*.*003*) and SBP (*P* = 0.013, *P* = 0.031) were increased compared to the control group. In prediabetes, TG (P = 0.021) and BUN (P = 0.014) were higher than in controls. HDL-c in prediabetes was lower (*P* = 0.032) than in controls. BMI increased in diabetics (*P* < 0.001) and prediabetics (*P* = 0.002) compared to controls. HOMA-IR, FBS, and HbA_1_c levels in diabetic and prediabetic groups were higher than in normal groups (*P* < 0.001). Insulin was increased in diabetics (*P* = 0.005) and prediabetics (*P* < 0.001).

### Oxidative stress parameters (PON-1, SOD-3, TAC, CAT, and MDA)

**Fig 3:**
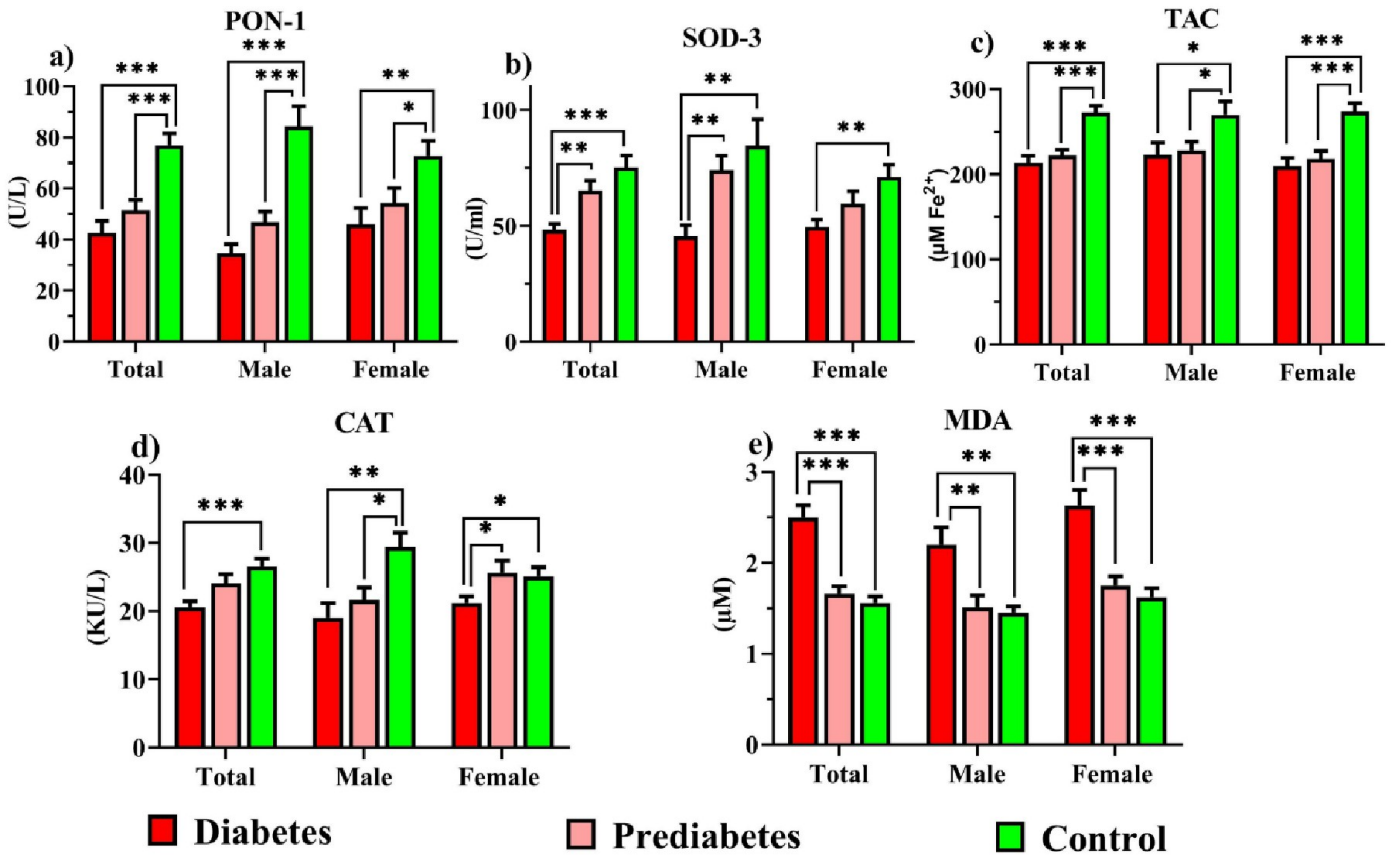
Comparison of PON-1, SOD-3, TAC, CAT, and MDA indices in study groups.

Fig 3 depicts OS parameters. The PON-1 enzyme activity and TAC were lower in diabetics and prediabetics than in controls (*P* < 0.001). Diabetics had lower SOD-3 activity than controls (*P* < 0.001) and prediabetics (*P* = 0.002). CAT activity declined in diabetics compared to controls (*P* < 0.001). MDA levels decreased more in the prediabetic and control groups than in the diabetes group (*P* < 0.001).

### Free fatty acids

**Fig 4:**
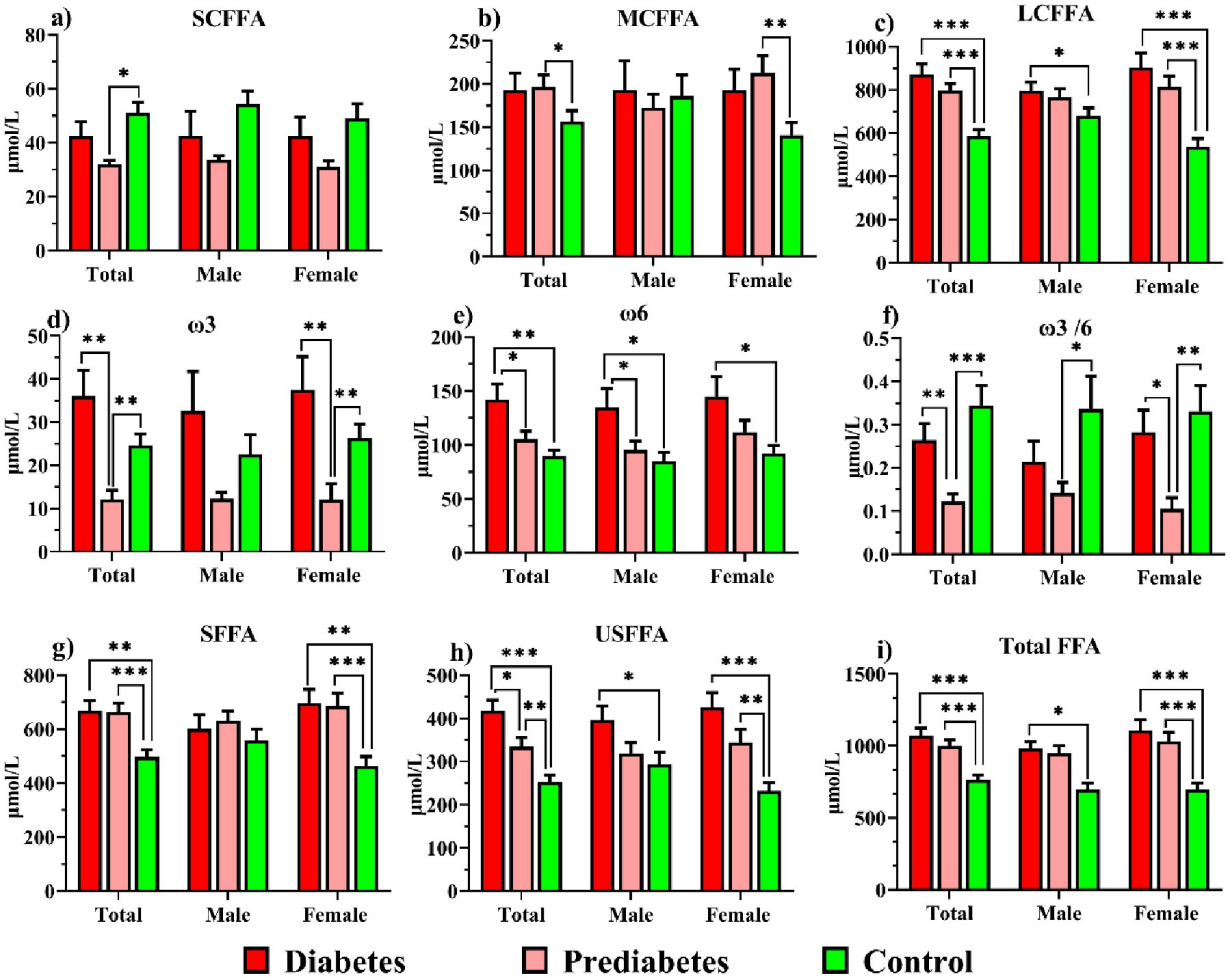
Comparison of SCFFA, MCFFA, LCFFA, ω3, ω6, ω3/6, SFFA, USFFA and total FFA in study groups.

FFA levels are shown in S2 Table in S1 File, and Fig 4 provides data about FFA levels based on length of chain and saturation. SCFFA in the prediabetics was reduced compared to controls (*P* = 0.01). MCFFA in prediabetics was higher than in controls (*P* = 0.036). ω3 levels were higher in individuals with diabetes (*P* = 0.001) and the control group (*P* = 0.005) than in prediabetes. ω6 was lower in control (*P* = 0.003) and prediabetes (*P* = 0.021) than diabetes. The ratio of ω3/6 decreased from prediabetes (1/8.1) to diabetes (1/3.7, *P* = 0.008) and control (1/2.99, *P* = 0.001). LCFFA, SFFA, USFFA, and total FFAs in diabetes and prediabetes were elevated in comparison to controls (*P* < 0.001).

### Correlation coefficient analysis

The correlation between the FFAs and the demographic information, HOMA-IR, and the OS parameters is shown in Table 1. The correlation coefficients vary from -0.396 to 0.557. SCFFA had an indirect correlation with BMI, HOMA-IR, and FBS and a direct correlation with TAC. LCFFA was positively related to BMI, HOMA-IR, HbA_1_C, FBS, and MDA and negatively related to TAC, PON-1, SOD-3, and CAT. SFFA had a direct correlation with HbA_1_C, FBS, and MDA and an indirect correlation with TAC and CAT. USFFA was positively correlated with BMI, HOMA-IR, HbA_1_C, FBS, and MDA and had a negative correlation with TAC, PON-1, and SOD-3. The ω3/ω6 ratio had a negative correlation to HOMA-IR, insulin, and FBS and a positive correlation to TAC. Total FFAs were positively related to BMI, HOMA-IR, HbA_1_C, FBS, and MDA and negatively related to TAC and CAT. Furthermore, gender had a direct correlation with BMI.

**Table 1:** Spearman’s Correlation between plasma FFAs and other parameters in the studied groups.

parameters	Gender	Antihypertensive medication	Age	BMI	HOMA-IR	insulin	HBA_1_C	FBS	TAC	MDA	PON-1	SOD-3	CAT
**SCFFA**	-0.119	-0.152	-0.078	-0.396**	-0.266*	-0.188	-0.232	-0.274*	0.081*	0.147	0.043	-0.015	-0.038
**MCFFA**	-0.047	-0.016	0.065	0.105	0.075	-0.003	0.079	0.163	-0.178	0.104	0.077	-0.037	-0.108
**LCFFA**	-0.037	0.162	0.061	0.199*	0.208*	0.161	0.371**	0.389**	-0.262**	0.336**	-0.279**	-0.208*	-0.281**
**SFFA**	-0.029	0.112	0.063	0.173	0.129	0.041	0.250*	0.330**	-0.301**	0.247**	-0.019	-0.120	-0.224*
**USFFA**	-0.035	0.134	0.010	0.219*	0.182*	0.130	0.373**	0.331**	-0.207*	0.363**	-0.358**	-0.209*	-0.179
**ω3/6**	0.005	-0.123	-0.230	-0.119	-0.388**	-0.371**	-0.125	-0.313*	0.182*	0.012	0.091	-0.003	0.171
**Total FFA**	-0.049	0.151	0.091	0.180*	0.181*	0.116	0.347**	0.357**	-0.335**	0.343**	-0.155	-0.147	-0.273**
**Gender**	1.000	0.118	-0.075	0.273**	0.095	0.067	-0.008	0.057	0.004	0.149	0.004	-0.096	0.061
**Antihypertensive medication**		1.000	0.216*	0.278**	0.199*	0.089	0.220*	0.213*	-0.157	0.161	-0.137	-0.141	-0.268**
**Age**			1.000	0.011	-0.078	0.064	0.105	0.132	-0.157	-0.039	-0.144	0.036	-0.030
**BMI**				1.000	0.265**	0.117	0.364**	0.361**	-0.210*	0.230*	-0.231**	-0.152	-0.123
**HOMA-IR**					1.000	0.666**	0.485**	0.557**	-0.192*	0.107	-0.282**	-0.151	-0.132

Spearman’s rho test was performed to examine the correlation between variables and the significance is as follows:

Correlation is significant at the 0.01 level (2-tailed) **

Correlation is significant at the 0.05 level (2-tailed) *

### Linear regression analysis

Linear regression was done to predict the effect of BMI, biochemical parameters, and OS on the FFAs. The results are shown in S3 Table in S1 File. BMI had a positive relationship with LCFFA, USFFA, and total FFAs and an inverse relationship with SCFFA. HOMA-IR was directly related to LCFFA, USFFA, and total FFAs and inversely related to ω3/ω6. Insulin had a positive relationship with USFFA and a negative relationship with ω3/6. HbA_1_C showed a positive relationship with LCFFA, USFFA, and total FFAs. FBS was directly related to MCFFA, LCFFA, SFFA, USFFA, and total FFAs and inversely related to SCFFA. TG was only directly related to LCFFA, USFFA, and total FFAs. PON-1 had an inverse relationship with LCFFA, USFFA, and FFAs. SOD-3 has a negative relationship with LCFFA and USFFA. CAT had an inverse relationship with LCFFA, SFFA, USFFA, and total FFAs. TAC had an inverse relationship with LCFFA, SFFA, USFFA, and total FFAs and a positive relationship with ω3/6. MDA was positively correlated with LCFFA, SFFA, USFFA, and total FFAs.

### Logistic regression analysis

Multinomial logistic regression was used to find out how T2DM (diabetes and prediabetes) was related to FFA levels. The results are shown in Table 2. Adjusted SCFFA significantly reduced T2DM severity in the third and fourth quartiles (≥ 38.7 μM). The MCFFA adjustment significantly increased the odds ratio for T2DM. The adjusted total FFAs (≥ 776.33 μM), LCFFA (≥ 613.58 μM), and SFFA (≥ 471.15 μM) significantly enhanced the severity of the T2DM. These parameters in the second, third, and fourth quarters increased the incidence of T2DM. Adjusted USFFA could significantly increase the severity of T2DM in the fourth quarter (≥ 398.68 μM).

**Table 2:** Association between T2DM incidence and FFAs by quartiles.

parameters	Crude				Adjusted			
	β	OR	CI-95%	*P*-value	β	OR	CI-95%	*P*-value
SCFFA	**-0.036**	**0.965**	**0.93 to 0.99**	**0.021**	-**0.035**	**0.965**	**0.938 to 0.996**	**0.027**
Q1<29.3 μM	Ref							
Q2(29.73–38.7) μM	0.486	1.62	0.23 to 11.46	0.62	0.271	1.31	0.148 to 11.64	0.80
Q3(38.7–55.88) μM	**-2.3**	**0.100**	**0.018 to 0.55**	**0.009**	**-2.57**	**0.074**	**0.011 to 0.55**	**0.011**
Q4>55.88 μM	**-1.67**	**0.188**	**0.036 to 0.96**	**0.033**	**-1.65**	**0.191**	**0.037 to 0.97**	**0.047**
MCFFA	0.004	1.004	1 to 1.008	0.051	**0.005**	**1.005**	**1 to 1.009**	**0.040**
Q1<108.34 μM	Ref							
Q2(108.34–168.13) μM	0.887	2.49	0.828 to 7.12	0.106	1.088	2.96	0.94 to 9.27	0.061
Q3(168.13–231.68) μM	0.626	1.87	0.696 to 5.02	0.214	0.561	1.75	0.61 to 4.99	0.294
Q4>231.68 μM	0.731	2.07	0.757 to 5.7	0.156	0.857	2.35	0.81 to 6.84	0.115
LCFFA	**0.007**	**1.007**	**1.004 to 1.010**	**<0.001**	**0.007**	**1.007**	**1.004 to 1.010**	**<0.001**
Q1<613.58 μM	Ref							
Q2(613.58–703.94) μM	**1.11**	**3.06**	**1.12 to 8.35**	**0.029**	**1.13**	**3.10**	**1.06 to 9.02**	**0.037**
Q3(703.94–856.64) μM	**1.69**	**5.46**	**1.88 to 15.88**	**0.002**	**1.64**	**5.16**	**1.65 to 16.13**	**0.005**
Q4>856.64 μM	**3.26**	**26.25**	**5.31 to 129.68**	**<0.001**	**3.24**	**25.65**	**4.69 to 132.62**	**<0.001**
SFFA	**0.005**	**1.005**	**1.002 to 1.007**	**<0.001**	**0.005**	**1.005**	**1.002 to 1.007**	**<0.001**
Q1<471.25 μM	Ref							
Q2(471.25–585.15) μM	**1.13**	**3.12**	**1.13 to 8.60**	**0.028**	**1.09**	**2.98**	**1.023 to 8.67**	**0.045**
Q3(585.15–727.58) μM	**1.28**	**3.61**	**1.29 to 10.15**	**0.015**	**1.27**	**3.57**	**1.20 to 10.64**	**0.022**
Q4>727.58 μM	**1.99**	**7.32**	**2.25 to 23.79**	**0.001**	**1.98**	**7.08**	**2.08 to 24.04**	**0.002**
USFFA	**0.008**	**1.008**	**1.004 to 1.012**	**<0.001**	**0.008**	**1.008**	**1.004 to 1.013**	**<0.001**
Q1<239.34 μM	Ref							
Q2(239.34–317.52) μM	0.305	1.35	0.51 to 3.60	0.54	0.224	1.25	0.445 to 3.52	0.671
Q3(317.52–398.68) μM	0.647	1.90	0.698 to 5.22	0.208	0.695	2	0.69 to 5.81	0.202
Q4>398.68 μM	**3.43**	**31**	**3.76 to 255.22**	**0.001**	**3.32**	**27.68**	**3.27 to 233.76**	**0.002**
Total FFA	**0.005**	**1.005**	**1.003 to 1.007**	**<0.001**	**0.005**	**1.005**	**1.003 to 1.007**	**<0.001**
Q1<776.33) μM	Ref							
Q2(776.33–895.58) μM	**1.11**	**3.03**	**1.21 to 8.28**	**0.030**	**0.964**	**2.63**	**1.11 to 9.39**	**0.041**
Q3(895.58–1064.76) μM	**1.29**	**3.64**	**1.29 to 10.26**	**0.014**	**1.21**	**3.37**	**1.14 to 9.92**	**0.027**
Q4>1064.76 μM	**3.16**	**23.75**	**4.77 to 118.04**	**<0.001**	**2.99**	**19.98**	**3.90 to 102.34**	**<0.001**

multinomial logistic regression test was performed to evaluate the severity of the T2DM (diabetes and prediabetes). The data were presented crude and adjusted with age, sex, and Antihypertensive medication. In addition, the data is expressed in quarters, with the first quarter being compared to other quarters as a reference.

Abbreviations: OR: Odds ratio, 95% CI: Confidence Interval, SCFFA: Short Chain Free Fatty Acid; MCFFA: Medium Chain Free Fatty Acid; LCFFA: Long Chain Free Fatty Acid; SFFA: Saturated Free Fatty Acid; USFFA: Unsaturated Free Fatty Acid; Total Free Fatty Acid (Total FFA).

### ROC curve analysis

**Fig 5:**
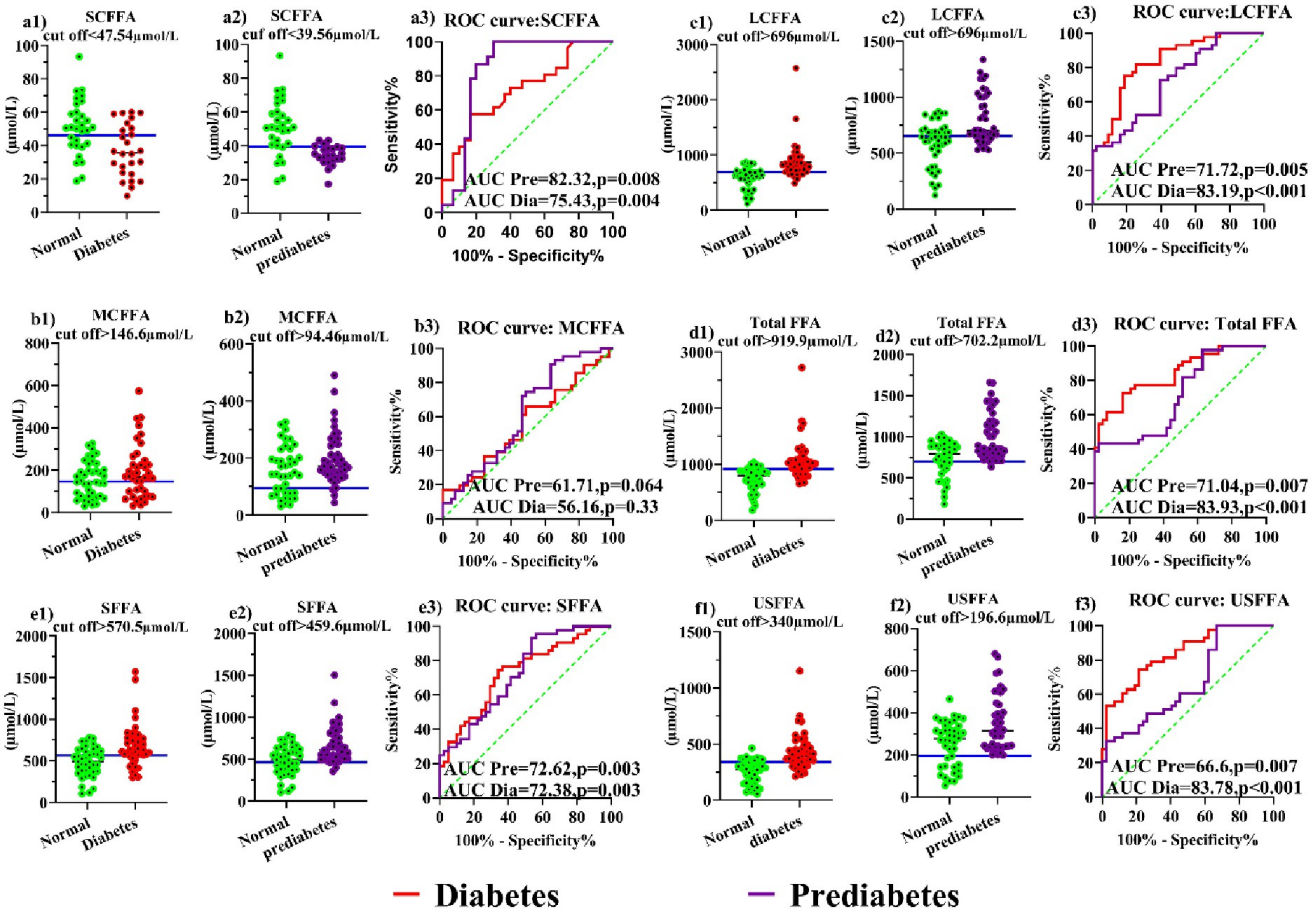
The ROC curve and the best cut-off for the FFAs in diabetes and prediabetes.

The ROC and AUC analyses of FFAs are used to find the best cut-off with the highest sensitivity and specificity for finding people with diabetes and prediabetes. Thus, an ideal ROC curve has an AUC of 1.0, and the results can be seen in Fig 5 and S4 Table in S1 File. Total FFA, USFFA, and LCFFA had the highest AUCs in diabetes. Also, in prediabetes, SCFFA, SFFA, and LCFFA had the highest AUC. AUC and the best cut-off point for FFAs in the detection of diabetes were obtained as follows: total FFA (AUC = 83.93, cut-off > 919.9 μM), USFFA (AUC = 83.78, cut-off > 340 μM), and LCFFA (AUC = 83.19, cut-off > 696 μM). Also, these were the best cut-off points for FFAs in finding people with prediabetes: SCFFA (AUC = 82.32, cut-off < 39.56 μM), SFFA (AUC = 72.62, cut-off > 459.6 μM), and LCFFA (AUC = 71.72, cut-off > 652.2 μM).

## Discussion

Albumin transports FFAs are non-esterified fatty acids in the blood. FFAs can cause IR by interfering with the phosphoinositide 3-kinase and insulin receptor tyrosine phosphorylation pathways. They can also cause inflammation by activating lipopolysaccharide (LPO) and TLR, increase oxidative phosphorylation, and cause OS [13, 14, 27, 34]. FFAR is affected by different types of FFAs and induces different effects [10], but the type and level of the FFA effect and its relationship to T2DM, IR, and OS have not been explored and are unclear. For this reason, for the first time, we evaluated the relationship between the type and levels of FFAs and different parameters, which were known as factors involved in the pathogenesis of T2DM. LCFFA, SFFA, USFFA, and total FFA levels were directly correlated with HOMA-IR, FBS, HbA_1_C, and MDA and inversely correlated with antioxidant parameters. Indeed, the adjusted LCFFA, SFFA, USFFA, and total FFA increased T2DM risk, while SCFFA and ω3/6 had an inverse relationship with BMI, HOMA-IR, and FBS and a direct relationship with TAC. Also, SCFFA adjustments reduced the development of T2DM. Total FFA, USFFA, and LCFFA had the greatest AUC in diabetes, whereas SCFFA had the highest AUC in prediabetes.

The human gut microbiota produces SCFFA from fiber and indigestible carbohydrates that reduce inflammation and OS [10]. The mechanism of action of plasma SCFFA involves reducing inflammation and enhancing thermogenesis in adipose tissue. It also works in the liver by increasing hepatic oxidation and decreasing inflammation, and it works in the pancreas by increasing insulin secretion [35]. Also, in dysbiotic microbiota imbalances, metformin may enhance the production of SCFFA by Lactobacillus and Escherichia coli [36]. SCFFAs (≥ 38.7 μM) have been shown to slow the development of T2DM, have a negative relationship with BMI, HOMA-IR, and FBS, and a positive relationship with TAC. In addition, this research revealed that SCFFA ≤ 39.56 μM had the best diagnosis for prediabetes.

The levels of MCFFAs were found to be higher in the prediabetic group, and they increased the risk of T2DM and had a positive correlation with FBS. A study by Marcal et al. found that rats fed MCFFA had lower glucose absorption in their muscles, lower PKB levels, and higher PKC levels in their pancreas. These changes may have contributed to the higher risk of T2DM [37]. In a study of diabetic mice by Rial et al., it was found that feeding animals with MCFFA increased thermogenesis in the liver and adipose tissue. MCFFAs as ligands can also be involved in activating PPARγ and reducing OS [38]. Various investigations into the effects of these fatty acids have shown different results, emphasizing the importance of further research on this topic.

In women, both the diabetes and prediabetic groups showed an increase in LCFFAs. However, in men, only the diabetic group exhibited high levels of LCFFAs. The LCFFA increases ROS by reducing electron current between complexes 1 and 3, and 3 and 4 [39]. Our results also show a direct relationship between LCFFAs and MDA, BMI, and HOMA-IR and an inverse relationship with antioxidant parameters. Most of the LCFFAs are derived from diet and circulating TG, which is directly related to these fatty acids [40], although not observed in our study. In our studies, because samples have been collected during fasting, lipolysis in adipose tissue and hepatocytes contributes to increasing LCFFAs [41].

Significant increases in SFFAs were observed in women with diabetes and prediabetes, while no significant differences were observed in men. SFFAs, by stimulating TLR, activate NFκB and JNK pathways and increase proinflammatory cytokines [42]. They also play a part in making more inflammasomes and interfering with the insulin signaling pathway [16], which makes the chance of getting T2DM higher. Huang et al. discovered that increased SFFAs and LPS increase HOMA-IR and the risk of T2DM incidence [43]. SFFAs induce apoptosis and decrease insulin production in pancreatic β-cells, increase mitochondrial dysfunction, and ultimately increase ROS production and OS [40]. They are positively related to MDA and negatively related to antioxidant parameters. In a study, the effect of saturated fatty acids (PA, C16:0) on muscle cells was investigated. It was observed that PA increased ROS production, induced apoptosis, and disrupted the insulin signaling pathway [44]. Mandal et al. demonstrated PA was higher in diabetes and had a positive correlation with HbA_1_C [12]. In this study, PA was increased in women in the diabetes group, but no difference was observed in men. In addition, PA, as a negative regulator of insulin function, was involved in inducing IR and inflammation [45]. Gaeini et al.’s meta-analysis found no connection between dietary total SFFA and the risk of T2DM [46], whereas our study revealed that an increase in SFFA contributes to T2DM. Kim et al. provided evidence that metformin effectively reduces SFFA and lipid accumulation, as well as reduces pro-inflammatory responses. These findings highlight the potential of metformin as a therapeutic approach for managing dyslipidemia and diabetes complications [47].

Based on this study, the USFFA was enhanced in the diabetic and prediabetic groups of women. However, in men, it was higher only in those with diabetes than in controls. Despite the positive effects of polyunsaturated fatty acids (PUSFAs), such as anti-inflammatory and anti-apoptotic properties, PUSFAs were prone to oxidation and the production of MDA and 4-HNE [26]. Studies have also shown that plasma PUSFAs may be involved in the production of O_2_^-^ via NADPH oxidase [39]. In a study by Pereira et al., it was found that SFFA and PUSFA reduce insulin sensitivity and amplify IR in the liver by PKC activation [48]. Li and colleagues’ analysis of the FFA profile in T2DM with cognition problems demonstrated that USFFA increases diabetes risk [49]. Although the effect of SFFA on IR is well known, the effect of USFFAs is contradictory. A study by Gehrmann et al. on β-cells discovered that SFFA (PA) damages and increases OS, but USFFAs >14 carbons protect against PA and do not produce OS [50]. Plötz et al. later discovered that SFFA (PA) and USFFA (oleic acid, C18:1) stimulate Caspase-3 in apoptosis and cell death, while oleic acid has no protective effect. FFAs >16 carbons are toxic to the pancreas, and fatty acids >14 carbons are involved in the induction of OS [51]. It seems that the lower the carbon fatty acid content and the smaller the saturation bond position, the more protective the fatty acids are and the less OS they cause.

The results displayed in ω3 were lower in prediabetics than in diabetics and controls. The administration of ω3 oil to diabetic rats resulted in improvements in their diabetes condition, characterized by a reduction in inflammation, decreased levels of FBS, HbA1c, lipid profile, and MDA, as well as an increase in the production of SCFFA [52]. Legrand found that consuming ω3 additionally contributes to lower inflammation by preventing inflammasome activation [16]. Amos et al. showed that ω3 increased insulin sensitivity in diabetic rats by increasing the antioxidant system [53]. ω3 acts as a ligand by binding to PPARγ to change the phenotype of macrophages to non-inflammatory (M2) and reduce inflammation [54]. Diabetes had higher levels of ω6 fatty acids than prediabetes and controls. This fatty acid is derived from diet, and studies have shown that these fatty acids are directly related to HOMA-IR and hyperinsulinemia, which play a role in increasing diabetes [55]. Kwon et al. demonstrated that ω6 fatty acids disrupted the insulin signaling pathway by promoting inflammation via the FOXO and JNK pathways, resulting in IR [56]. The ω3/6 fatty acid ratio was lower in prediabetics (1/8.1) than in controls (1/2.99) and diabetics (1/3.7). ω3/6 was inversely related to HOMA-IR, insulin, and FBS and directly related to TAC. In diabetics, the ratio of ω3/6 (1/1 to 1/5) improved insulin function by increasing the expression of uncoupling protein 1 in adipose tissue, decreasing FBS, cholesterol, TG, and LDL-c, and increasing glucose tolerance [57]. The ratio of ω3/6 in diabetics (1/13) decreased compared to the control group (1/4) based on the results achieved by Shetty et al. [58]. Castro et al. showed that ω3 fatty acid levels were higher in prediabetes, and the ratio of ω3/6 fatty acids was lower in the control population [59]. Alhazmi et al. showed that higher levels of ω6 as well as ω3 are associated with an increased risk of diabetes [60]. Diabetics tend to attenuate the intensity and manage the T2DM by enhancing their intake of ω3 fatty acids, while prediabetics have limited control over ω3 levels, which can accelerate the progression of the T2DM.

Total FFA in the diabetic and prediabetic groups of women increased compared to controls, but in men, it only increased in diabetes. Total FFAs positively correlated with BMI, HOMA-IR, and MDA and were inversely correlated with TAC and CAT. Like LCFFA, SFFA, and USFFAs, total FFAs underwent more severe changes in females than in males. Since the BMI went up a lot in the female patient groups but didn’t change much in the male patient groups, it can be said that weight gain and BMI play a key role in increasing the fatty acids. This was linked to the growth of IR because it caused diacylglycerol to be made, PKC, JNK, SOCS to be activated, and inflammatory cytokines to be released. It also caused OS to begin [13]. Total FFAs cause insulin to lose its function by increasing β-oxidation and ATP/AMP growth, resulting in impaired glucose metabolism, inhibition of glycolysis, stimulation of gluconeogenesis in the liver, and decreased glucose uptake and glycogen synthase activity in muscle [11]. Spiller et al. found that total FFAs were higher in diabetics and prediabetics than in controls. Total FFAs were directly related to IR. When IR hits adipose tissue and hepatocytes, it speeds up lipolysis, which raises the amount of FFA in the plasma [41]. But in this study, we evaluated FFA in plasma without a source of release. Also, measuring fatty acids as one of the methods of diagnosing the T2DM has high sensitivity and specificity [19]. Ma et al. discovered that docosahexaenoic acid (DHA, C22:6) has a high potential to predict susceptibility to T2DM (AUC = 80.3) [61]. Our research also discovered that total FFAs had the best diabetes diagnosis (AUC = 83.93, cut-off > 919.9 μM). Huang et al. reported that higher total FFA (FFAs > 623.20 mol/L) contributed to T2DM through promoting hs-CRP, inflammation, and HOMA-IR [43]. The study conducted by Gregorio et al. indicates that metformin reduces plasma levels of total FFA, particularly in individuals with a high waist-hip ratio. This data highlights the potential of metformin as an effective treatment approach [62]. However, further investigation is required to compare the dosage of metformin with the normal administration.

The strengths of this study were categorizing patients into diabetics in early-stage treatment with metformin and newly diagnosed prediabetes. Measuring and analyzing FFAs according to size and grade of saturation, determining the levels of FFAs as a risk factor, and identifying the diagnosis of diabetes and prediabetes based on FFA levels. On the other hand, our study has several limitations. Despite measuring FFA levels, it appears that the place of residence, lifestyle, calorie and fat intake, intestinal microbiota activity, FFAR expression, albumin level, and effect of lipolysis in adipose tissue and hepatocytes must be considered. Also, if we increase the sample size, measure other parameters of OS, conduct other methods for assaying IR such as the glucose clamp test, and resolve the above limitations, the results will be the most valid and properly evaluate the role of FFAs in T2DM. The results of this study have given us an insight into the role of FFAs in T2DM and OS, as well as their use to diagnose diabetes and prediabetes. Nutritionists and researchers in the field of metabolic and inflammatory diseases can design and implement their studies by considering the role of FFAs. While research has demonstrated the advantageous impact of metformin in lowering FFAs levels, its specific mechanism and effects on different types of FFAs remain uncertain and require further investigation. As well as, we will conduct a clinical trial to survey the effect of metformin and others diabetes drugs on FFA levels in diabetes and their association with IR parameters and OS.

## Conclusion

The study showed that total FFA (≥ 776 μM), LCFFA (≥ 613 μM), SFFA (≥ 471 μM), and USFFA (≥ 398 μM) raised the risk of T2DM by raising OS and HOMA-IR. The levels of these fatty acids showed a greater increase in females compared to males. Raising BMI performed a vital function in developing these FFAs. The risk of diabetes, OS, and HOMA-IR is reduced by SCFFA (≥ 38.7 μM). Furthermore, for the diagnosis of prediabetes and diabetes, SCFFAs and total FFAs can be used, respectively.

## Supporting information

S1 File S1-S4 Tables. (DOCX)

## Abbreviations

ALT: Alanine Transaminase
AST: Aspartate Transaminase
AUC: Area Under Curve
BMI: Body mass index
BUN: Blood Urea Nitrogen
CAT: Catalase
Cr: Creatinine
FBS: Fasting Blood Sugar
FFAR: Free Fatty Acid Receptor
FFAs: Free Fatty Acids
HbA_1_C: Glycated Hemoglobin
HDL-c: High-Density Lipoprotein cholesterol
HOMA-IR: Homeostatic Model Assessment for Insulin Resistance
IFG: Impaired Fasting Glucose
IR: Insulin Resistance
IR: Insulin Resistance
JNK: c-Jun N-terminal kinase
LCFFA: Long Chain Free Fatty Acid
LDL-c: Low-Density Lipoprotein cholesterol
MDA: Malondialdehyde
OS: Oxidative Stress
PA: Palmitic Acid
PKC: Protein kinase C
PON-1: Paraoxonase-1
ROC: Receiver Operating Characteristic
SBP: systolic blood pressure
SCFFA: Short Chain Free Fatty Acid
SFFA: Saturated Free Fatty Acid
SOD-3: Superoxide dismutase-3
T2DM: Type 2 diabetes mellitus
TAC: Total Antioxidant Capacity
TC: Total Cholesterol
TG: Triglycerides
TLR: Toll-like receptor
Total FFA: Total Free Fatty Acid
UA: Uric Acid
USFFA: Unsaturated Free Fatty Acid
