# Supplementary material for: Relationship between types and levels of free fatty acids, peripheral insulin resistance, and oxidative stress in T2DM: A case-control study
Source: PLoS One. 2024 Aug 12;19(8):e0306977. doi: 10.1371/journal.pone.0306977 (PMC11318896; doi:10.1371/journal.pone.0306977)
Supplement: S1 File — (DOCX) [file pone.0306977.s001.docx]

**S1 Table:** **Demographic data and biochemical markers of diabetic and prediabetic patients**

| Groups  Variables | | Sample | Diabetes  (n=60) | Prediabetes  (n=60) | Control  (n=60) | *P*-value |
| --- | --- | --- | --- | --- | --- | --- |
| Gender  (n (%)) | | Male | 25(31.25) | 28(35) | 27(33.75) | 0.66 |
|  |  | Female | 35(35) | 32(32) | 33(33) |  |
|  |  | Total | 60 | 60 | 60 |  |
| Age (years) | | Male | 49.92±1.96 | 53.82±1.91 | 51.6±1.52 | 0.32 |
|  |  | Female | 52.32±1.08 | 52.07±1.24 | 49.25±1.01 | 0.10 |
|  |  | Total | 51.61±0.96 | 52.75±1.05 | 50.06±0.85 | 0.147 |
| BMI  (Kg/m^2^) | | Male | 25.28±0.8 | 25.25±0.73 | 23.66±0.7 | 0.249 |
|  |  | Female | 28.15±0.67 a*** | 27.46±0.6  b** | 24.5±0.43 | **<0.001** |
|  |  | Total | 27.31±0.56 a*** | 26.58±0.49 b** | 24.20±0.39 | **<0.001** |
| Antihypertensive medication  (n (%)) | | Male | 7(41.17) | 8(47.05) | 2(11.76) | **0.003** |
|  |  | Female | 20(51.28) | 14(35.89) | 5(12.82) |  |
|  |  | Total | 27(48.21)  a** | 22(39.28)  b* | 7(12.5) |  |
| Blood pressure (D-S) | **DBP**  **(mm Hg)** | Male | 79.58±5.67 | 85.29±0.91 | 83.8±0.84 | 0.36 |
|  |  | Female | 86±0.58  a** | 86.48±0.49 b** | 83.36±0.61 | **0.001** |
|  |  | Total | 84.16±1.68 | 86.02±1.46 | 83.52±1.48 | 0.217 |
|  | **SBP**  **(mm Hg)** | Male | 129.2±2 | 130.17±1.83 | 125.73±1.27 | 0.153 |
|  |  | Female | 131.2±1.30  a* | 130.14±1.35 | 126.88±1.14 | **0.056** |
|  |  | Total | 130.66±1.089 a* | 130.15±1.078 b* | 126.45±0.85 | **0.009** |
| TC (mg/dl) | | Male | 156.16±8.03 | 148.57±9.97 | 149.53±7.18 | 0.802 |
|  |  | Female | 151.79±8.62 | 167.12±9.36 | 157.62±4.44 | 0.377 |
|  |  | Total | 153.25±6.28 | 160.28±7.03 | 154.78±3.82 | 0.673 |
| TG (mg/dl) | | Male | 155.66±20.51 | 143.35±13.77 | 135.61±14.46 | 0.861 |
|  |  | Female | 143.22±11.02 | 163.3±13.4  b* | 122.12±7.93 | 0.085 |
|  |  | Total | 147.61±10.03 | 155.75±9.83 b* | 126.86±7.19 | 0.138 |
| HDL-c (mg/dl) | | Male | 42.66±3.4 | 38.27±2.86 | 45±2.89 | 0.348 |
|  |  | Female | 45.36±2.21 | 41.5±1.99 | 48.25±2.76 | 0.141 |
|  |  | Total | 44.41±1.85 | 40.35±1.63  b* | 47.32±2.12 | **0.04** |
| LDL-c (mg/dl) | | Male | 82.66±6.47 | 88.36±8.47 | 79.5±6.63 | 0.711 |
|  |  | Female | 79.42±8.42 | 92.65±9.27 | 83.3±4.93 | 0.472 |
|  |  | Total | 80.60±5.79 | 91.12±6.61 | 82.21±3.94 | 0.366 |
| BUN (mg/dl) | | Male | 30±1.92 | 33.64±4.83 | 23.53±1.68 | 0.109 |
|  |  | Female | 28.14±1.82 | 30.71±2.63 | 26.38±1.64 | 0.355 |
|  |  | Total | 28.74±1.37 | 32.17±2.71  b* | 25.19±1.19 | **0.034** |
| Cr (mg/dl) | | Male | 1.04±0.07 | 1.43±0.36 | 1.02±0.05 | 0.50 |
|  |  | Female | 0.90±0.035 | 0.89±0.036 | 0.91±0.024 | 0.84 |
|  |  | Total | 0.95±0.036 | 1.15±0.18 | 0.96±0.28 | 0.278 |
| Uric Acid (mg/dl) | | Male | 4.24±0.09 | 5.32±0.59 | 5.22±0.49 | 0.306 |
|  |  | Female | 4.32±0.28 | 4.53±0.14 | 4.48±0.23 | 0.735 |
|  |  | Total | 4.29±0.18 | 4.58±0.24 | 4.73±0.23 | 0.238 |
| SGOT-AST (U/L) | | Male | 25.71±1.7 | 24.11±2.0 | 22.16±2.91 | 0.57 |
|  |  | Female | 26.08±1.51 | 26.75±1.99 | 23.42±1.37 | 0.283 |
|  |  | Total | 25.94±1.11 | 25.35±1.41 | 23.12±1.23 | 0.227 |
| SGPT-ALT (U/L) | | Male | 28.42±2. | 25.66±2.3 | 23±3.42 | 0.399 |
|  |  | Female | 31.16±2.06 | 26.37±2.14 | 26.31±2.36 | 0.29 |
|  |  | Total | 30.15±1.52 | 26±1.54 | 25.52±1.96 | 0.143 |

Parameters are presented as mean ± SEM and number (%). One-way ANOVA/Kruskal-Wallis tests were used to analyze quantitative data, and Chi-square test was used to analyze qualitative data.

a: Comparison between the diabetes and control groups; b: Comparison between the prediabetes and control groups. The significance level is as: * *P* < 0.05, ** *P* < 0.01, *** *P* < 0.001.

Abbreviations: BMI: Body Mass Index; DBP: Diastolic Blood Pressure; SBP: Systolic Blood Pressure. TC: Total Cholesterol; TG: Triglycerides; HDL-c: High-Density Lipoprotein-cholesterol; LDL-c: Low-Density Lipoprotein-cholesterol; ALT: Alanine Transaminase; AST: Aspartate Transaminase; CR: Creatinine; BUN: Blood Urea Nitrogen.

**S2 Table: Free fatty acid levels in diabetic and prediabetic patients**

| Groups  Variables | Sample | Diabetes  Mean ±SE | Prediabetes  Mean ±SE | Control  Mean ±SE | *p*-value |
| --- | --- | --- | --- | --- | --- |
| Caprylic acid(C8:0)  (μmol/L) | Male | 39.83±8.77 | 33.67±1.53 | 54.39±4.75 | 0.057 |
|  | Female | 44.28±7.88 | 31.09±2.22 | 48.83±5.55 | 0.135 |
|  | Total | 42.71±5.82 | 32.01±1.52  **b*** | 51.01±3.82 | **0.013** |
| Capric acid(C10:0)  (μmol/L) | Male | 174.63±23.7 | 139±10.68 | 138.74±14.76 | 0.301 |
|  | Female | 157.88±8.92 | 164.96±14.87 | 124.03±9.25 | 0.274 |
|  | Total | 161.58±15.39 | 154.93±10.13 | 130.16±8.12 | 0.42 |
| Lauric acid(C12:0)  (μmol/L) | Male | 61.23±9.71 | 60.69±11.35 | 93.52±13.93 | 0.199 |
|  | Female | 63.71±13.61 | 68.16±8.38 | 82.29±8.19 | 0.327 |
|  | Total | 62.91±9.63  **a*** | 65.33±6.68  **b*** | 86.33±7.19 | **0.015** |
| Myristic acid(C14:0)  (μmol/L) | Male | 126.28±5.65 | 119.5±8.68 | 107.81±9.1 | 0.392 |
|  | Female | 132.81±22.22 | 112.84±8.28 | 104.45±10.84 | 0.549 |
|  | Total | 131.11±16.41 | 115.47±6.02 | 105.52±7.82 | 0.341 |
| Myristoleic acid(C14:1)  (μmol/L) | Male | 119.17±13.28 | 106.4±12.07 | 86.74±10.18 | 0.177 |
|  | Female | 126.49±22.74 | 111.15±10.48  **b*** | 80.02±10.09 | **0.046** |
|  | Total | 124.37±16.5  **a*** | 109.32±7.85  **b*** | 82.63±7.25 | **0.012** |
| Palmitic acid(C16:0)  (μmol/L) | Male | 257.73±23.89 | 220.53±14.75 | 222.06±14.79 | 0.454 |
|  | Female | 301.31±23.64  **a**** | 237.14±21.6 | 201.99±9.1 | **<0.001** |
|  | Total | 288.43±18.9  **a**, c*** | 230.71±14.35 | 211.62±8.17 | **0.001** |
| Palmitoleic acid(C16:1)  (μmol/L) | Male | 19.45±3.9  **a*** | 15.03±3.13  **b**** | 36.43±6.86 | **0.005** |
|  | Female | 21.59±2.11  **a*** | 19.1±2.47  **b*** | 35.55±6.59 | **0.07** |
|  | Total | 21.01±1.85  **a**** | 17.51±1.94  **b***** | 35.93±4.71 | **0.001** |
| Stearic acid(C18:0)  (μmol/L) | Male | 94.1±1.84 | 95.25±5.48 | 86.17±5.25 | 0.372 |
|  | Female | 111.63±16.81 | 96.97±7.02 | 83.11±5.46 | 0.239 |
|  | Total | 106.15±11.8 | 96.3±4.75 | 84.2±3.94 | 0.135 |
| Oleic acid(C18:1n9c)  (μmol/L) | Male | 123.28±22.18 | 105.56±12.1  **b*** | 158.66±19.01 | 0.08 |
|  | Female | 117.53±7.45 | 117.44±12.43 | 99.87±9.26 | 0.41 |
|  | Total | 119.36±8.52 | 112.74±74.67 | 122.63±10.49 | 0.615 |
| Linoleic acid(C18:2n6c)  (μmol/L) | Male | 134.78±17.44  **a*, c*** | 95.26±8.28 | 84.93±8.31 | 0.067 |
|  | Female | 144.91±18.22  **a*** | 112.05±10.94 | 92.27±7.19 | 0.104 |
|  | Total | 142.19±14.04  **a**, c*** | 105.41±7.41 | 89.67±5.45 | 0.008 |
| Alpha-linolenic acid(C18:3n3)  (μmol/L) | Male | 32.62±9.09  **a*** | 12.22±1.52 | 22.54±4.57 | 0.052 |
|  | Female | 37.42±7.72  **c**** | 12.08±3.64  **b**** | 26.37±3.21 | **0.011** |
|  | Total | 36±6  **c**** | 12.14±2.09  **b**** | 24.67±2.66 | **0.001** |
| Arachidic acid  (μmol/L) | Male | 28.26±4.17 | 20.2±4.17 | 28.76±5.07 | 0.126 |
|  | Female | 44.01±11.95 | 35.46±8 | 21.37±3.85 | 0.705 |
|  | Total | 39.16±8.41 | 30.22±5.54 | 24.33±3.10 | 0.743 |

Parameters are presented as mean ± SEM. One-way ANOVA/Kruskal-Wallis tests with post-hoc Tukey/Mann-Whitney U tests were used to analyze data.

a: Comparison between the diabetes and control groups; b: Comparison between the prediabetes and control groups; and c: Comparison between the diabetes and prediabetes groups. The significance level is as: * *P* < 0.05, ** *P* < 0.01, *** *P* < 0.001.

**S3 Table: Predictors of plasma concentrations of FFAs, Linear regression analyses.**

| **variable** | **SCFFA** | | | **MCFFA** | | | **LCFFA** | | | **SFFA** | | | **USFFA** | | | **ω 3/6** | | | **Total FFA** | | |
| --- | --- | --- | --- | --- | --- | --- | --- | --- | --- | --- | --- | --- | --- | --- | --- | --- | --- | --- | --- | --- | --- |
|  | **Slope** | **95% CI** | ***P*-value** | **Slope** | **95% CI** | ***P*-value** | **Slope** | **95% CI** | ***P*-value** | **Slope** | **95% CI** | ***P*-value** | **Slope** | **95% CI** | ***P*-value** | **Slope** | **95% CI** | ***P*-value** | **Slope** | **95% CI** | ***P*-value** |
| **BMI** | **-1.99** | **-3.53**  **-0.44** | **0.01** | 2.13 | -3.19  7.46 | 0.43 | **16.29** | **1.85**  **30.68** | **0.02** | 8.92 | -3.01  20.86 | 0.14 | **9.52** | **1.5**  **17.55** | **0.02** | -0.006 | -0.02  0.007 | 0.37 | **16.26** | **1.15**  **32.6** | **0.045** |
| **HOMA-IR** | -0.261 | -1.77  1.25 | 0.73 | -0.182 | -5.25  4.89 | 0.94 | **16.25** | **2.82**  **29.69** | **0.01** | 6.07 | -5.29  17.44 | 0.29 | **7.83** | **0.411**  **15.25** | **0.03** | **-0.017** | **-0.03**  **-0.004** | **0.012** | **16.56** | **1.59**  **31.88** | **0.035** |
| **insulin** | -0.025 | -0.485  0.435 | 0.91 | -0.79 | -2.5  0.926 | 0.36 | **4.61** | **-0.007**  **9.24** | **0.050** | 0.15 | -3.76  4.06 | 0.94 | **2.71** | **0.181**  **5.24** | **0.03** | **-0.005** | **-0.009**  **-0.002** | **0.007** | 4.13 | -1.16  9.43 | 0.125 |
| **HbA_1_c** | -2.06 | -7.13  3.01 | 0.41 | 6.53 | -10.88  23.94 | 0.45 | **62.08** | **16.18**  **107.98** | **0.009** | 34.34 | -3.17  71.85 | 0.07 | **42.79** | **17.36**  **67.96** | **0.001** | -0.007 | -0.055  0.041 | 0.75 | **64.68** | **12.5**  **116.68** | **0.016** |
| **FBS** | **-0.157** | **-0.309**  **-0.004** | **0.04** | **0.547** | **0.034**  **1.06** | **0.037** | **2.29** | **0.938**  **3.64** | **0.001** | **1.93** | **0.804**  **3.05** | **0.001** | **1.3** | **0.522**  **2.09** | **0.001** | -0.001 | -0.002  0.001 | 0.20 | **2.64** | **1.1**  **4.18** | **0.001** |
| **TG** | 0.014 | -0.067  0.095 | 0.72 | 0.271 | -0.101  0.643 | 0.15 | **1.15** | **0.389**  **1.91** | **0.003** | 0.759 | -0.032  1.54 | 0.06 | **0.596** | **0.142**  **1.05** | **0.01** | 0.000 | -0.002  0.001 | 0.40 | **1.55** | **0.586**  **2.51** | **0.002** |
| **PON-1** | -0.028 | -0.177  0.121 | 0.70 | -0.033 | -0.599  0.532 | 0.9 | **-1.79** | **-3.25**  **-0.332** | **0.017** | -0.788 | -2.02  0.45 | 0.21 | **-1.26** | **-2.05**  **-0.48** | **0.002** | 0.000 | -0.001  0.002 | 0.689 | **-1.75** | **-3.43**  **-0.079** | **0.04** |
| **SOD-3** | -0.024 | -0.219  0.170 | 0.80 | -0.077 | -0.752  0.598 | 0.82 | **-2.32** | **-4.25**  **-0.396** | **0.019** | -1.05 | -2.66  0.564 | 0.2 | **-1.52** | **-2.54**  **-0.497** | **0.004** | -8.44 | -0.002  0.002 | 0.937 | -1.76 | -3.95  0.418 | 0.11 |
| **CAT** | -0.079 | -0.851  0.963 | 0.83 | -1.12 | -3.69  1.44 | 0.387 | **-10.72** | **-18.16**  **-3.29** | **0.005** | **-7.25** | **-13.09**  **-1.41** | **0.01** | **-4.69** | **-8.73**  **-0.651** | **0.02** | 0.005 | -0.002  0.011 | 0.166 | **-11.53** | **-19.7**  **-3.43** | **0.006** |
| **TAC** | 0.07 | -0.095  0.109 | 0.12 | -0.317 | -0.638  0.003 | 0.052 | **-1.36** | **-2.25**  **-0.485** | **0.003** | **-1.2** | **-1.92**  **-0.472** | **0.001** | **-0.744** | **-1.25**  **-0.237** | **0.004** | **0.011** | **0.001**  **0.002** | **0.042** | **-1.77** | **-2.74**  **-0.765** | **0.001** |
| **MDA** | 4.62 | -1.79  11.04 | 0.154 | 18.9 | -4.78  42.58 | 0.117 | **96.24** | **32.84**  **159.64** | **0.003** | **63.79** | **10.14**  **117.46** | **0.02** | **69.09** | **34.98**  **130.21** | **<0.001** | 0.06 | -0.003  0.124 | 0.06 | **123.81** | **52.62**  **195** | **0.001** |

A linear regression test was carried out to investigate the effect of OS and biochemical and demographic data (independent variables) on different types of fatty acids (dependent variables), and the significance level was *P* < 0.05.

Abbreviations: 95% CI: Confidence Interval.

**S4 Table: AUC and cut-off for FFAs in diabetes and prediabetes**

| Parameters | Case | AUC | Sensitivity | Specificity | Cut Off | 95% CI | *P*-value |
| --- | --- | --- | --- | --- | --- | --- | --- |
| SCFFA | Diabetes | 75.43 | 75 | 65.22 | <47.54 µM | 0.60-0.89 | **0.004** |
|  | prediabetes | 82.32 | 100 | 78.26 | <39.56 µM | 0.69 to 0.97 | **0.008** |
| MCFFA | Diabetes | 56.16 | 65.85 | 51.22 | >146.6 µM | 0.43-0.68 | 0.33 |
|  | prediabetes | 61.71 | 93.02 | 34.15 | >94.56 µM | 0.49-0.73 | 0.064 |
| LCFFA | Diabetes | 83.19 | 81.82 | 74.42 | >696 µM | 0.74-0.91 | **<0.001** |
|  | prediabetes | 71.72 | 72.73 | 60.47 | >652.2 µM | 0.61-0.82 | **0.005** |
| SFFA | Diabetes | 72.38 | 74.42 | 65.85 | >570.5 µM | 0.62-0.83 | **0.003** |
|  | prediabetes | 72.62 | 93.18 | 46.34 | >459.6 µM | 0.61-0.83 | **0.003** |
| USFFA | Diabetes | 83.78 | 72.09 | 78.57 | >340 µM | 0.75-0.91 | **<0.001** |
|  | prediabetes | 66.6 | 94.45 | 33.33 | >196.6 µM | 0.55-0.78 | **0.008** |
| Total FFA | Diabetes | 83.93 | 72.73 | 83.72 | >919.9 µM | 0.75-0.92 | **<0.001** |
|  | prediabetes | 71.04 | 97.73 | 37.21 | >702.2 µM | 0.60-0.81 | **0.007** |

AUC assessment by ROC curve analysis and the best cut-off for FFAs calculated with the highest sensitivity and specificity in the diabetes and prediabetes groups.
